# *dbl-1*/TGF-β and *daf-12*/NHR Signaling Mediate Cell-Nonautonomous Effects of *daf-16*/FOXO on Starvation-Induced Developmental Arrest

**DOI:** 10.1371/journal.pgen.1005731

**Published:** 2015-12-11

**Authors:** Rebecca E. W. Kaplan, Yutao Chen, Brad T. Moore, James M. Jordan, Colin S. Maxwell, Adam J. Schindler, L. Ryan Baugh

**Affiliations:** Department of Biology, Duke University, Durham, North Carolina, United States of America; University of California San Francisco, UNITED STATES

## Abstract

Nutrient availability has profound influence on development. In the nematode *C*. *elegans*, nutrient availability governs post-embryonic development. L1-stage larvae remain in a state of developmental arrest after hatching until they feed. This “L1 arrest” (or "L1 diapause") is associated with increased stress resistance, supporting starvation survival. Loss of the transcription factor *daf-16*/FOXO, an effector of insulin/IGF signaling, results in arrest-defective and starvation-sensitive phenotypes. We show that *daf-16*/FOXO regulates L1 arrest cell-nonautonomously, suggesting that insulin/IGF signaling regulates at least one additional signaling pathway. We used mRNA-seq to identify candidate signaling molecules affected by *daf-16*/FOXO during L1 arrest. *dbl-1*/TGF-β, a ligand for the Sma/Mab pathway, *daf-12*/NHR and *daf-36*/oxygenase, an upstream component of the *daf-12* steroid hormone signaling pathway, were up-regulated during L1 arrest in a *daf-16*/FOXO mutant. Using genetic epistasis analysis, we show that *dbl-1*/TGF-β and *daf-12*/NHR steroid hormone signaling pathways are required for the *daf-16*/FOXO arrest-defective phenotype, suggesting that *daf-16*/FOXO represses *dbl-1*/TGF-β, *daf-12*/NHR and *daf-36*/oxygenase. The *dbl-1*/TGF-β and *daf-12*/NHR pathways have not previously been shown to affect L1 development, but we found that disruption of these pathways delayed L1 development in fed larvae, consistent with these pathways promoting development in starved *daf-16*/FOXO mutants. Though the *dbl-1*/TGF-β and *daf-12*/NHR pathways are epistatic to *daf-16*/FOXO for the arrest-defective phenotype, disruption of these pathways does not suppress starvation sensitivity of *daf-16*/FOXO mutants. This observation uncouples starvation survival from developmental arrest, indicating that DAF-16/FOXO targets distinct effectors for each phenotype and revealing that inappropriate development during starvation does not cause the early demise of *daf-16*/FOXO mutants. Overall, this study shows that *daf-16*/FOXO promotes developmental arrest cell-nonautonomously by repressing pathways that promote larval development.

## Author Summary

Animals must cope with feast and famine in the wild. Environmental fluctuations require a balancing act between development in favorable conditions and survival during starvation. Disruption of the pathways that govern this balance can lead to cancer, where cells proliferate when they should not, and metabolic diseases, where nutrient sensing is impaired. In the roundworm *Caenorhabditis elegans*, larval development is controlled by nutrient availability. Larvae are able to survive starvation by stopping development and starting again after feeding. Stopping and starting development in this multicellular animal requires signaling to coordinate development across tissues and organs. How such coordination is accomplished is poorly understood. Insulin/insulin-like growth factor (IGF) signaling governs larval development in response to nutrient availability. Here we show that insulin/IGF signaling activity in one tissue can affect the development of other tissues, suggesting regulation of additional signaling pathways. We identified two pathways that promote development in fed larvae and are repressed by lack of insulin/IGF signaling in starved larvae. Repression of these pathways is crucial to stopping development throughout the animal during starvation. These three pathways are widely conserved and associated with disease, suggesting the nutrient-dependent regulatory network they comprise is important to human health.

## Introduction

*C*. *elegans* L1-stage larvae must feed upon hatching in order to exit developmental arrest. Larvae in L1 arrest have increased stress resistance and can survive for weeks, initiating postembryonic development when food is available [1]. *C*. *elegans* larvae can also arrest development during the dauer stage, an alternative to the third larval stage that forms in response to crowding, nutrient stress or high temperature [2]. Unlike dauer formation, L1 arrest is an acute starvation response without an alternative developmental program, making it an excellent model for nutritional control of development.

The insulin/insulin-like growth factor (IGF) signaling pathway is a key regulator of L1 arrest, mediating the systemic response to nutrient availability [3,4]. In fed larvae, insulin-like peptides act through the insulin/IGF receptor DAF-2/InsR, activating a conserved PI3K cascade to repress function of the forkhead-type transcription factor DAF-16/FOXO [5–8]. In starved larvae, DAF-16/FOXO is active and promotes L1 arrest [3]. A variety of genome-wide gene expression analyses have been published for *daf-16*/FOXO, and a meta-analysis of them identified thousands of genes whose expression is positively or negatively affected by *daf-16*/FOXO activity [9]. This study also identified the transcription factor PQM-1 as a mediator of *daf-16*-dependent effects on gene expression. However, these experiments were done in young adult animals and with a *daf-2*/InsR mutant background to activate DAF-16/FOXO as opposed to starvation. DAF-16/FOXO target genes that promote L1 arrest are largely unknown. *daf-16*/FOXO activates the cyclin-dependent kinase inhibitor *cki-1*/p27 and represses the developmental timing microRNA *lin-4* [3], but whether such regulation is direct is unclear.

The insulin/IGF pathway is pleiotropic, serving as a key regulator in dauer formation, lifespan, associative learning, and stress resistance [5,10–15]. *daf-2*/InsR and *daf-16*/FOXO affect lifespan and dauer formation cell-nonautonomously [16–20]; that is, *daf-16*/FOXO activity in limited tissues affects the phenotype of the entire organism. The insulin/IGF pathway is highly conserved, and it also affects lifespan cell-nonautonomously in *Drosophila* and mice [21–24]. *daf-16*/FOXO activity in one tissue has been shown to affect *daf-16*/FOXO activity in other tissues through feedback regulation, termed FOXO-to-FOXO signaling [25–27]. Insulin/IGF receptor cell-nonautonomy could result from FOXO-to-FOXO signaling, since FOXO is still present in the affected tissues. However, FOXO cell-nonautonomy is inconsistent with FOXO-to-FOXO signaling because in this experimental scenario FOXO is not present in the affected tissues. Instead, FOXO cell-nonautonomy suggests FOXO regulates an additional signaling pathway to affect cells where it is not present.

The *daf-12*/nuclear hormone receptor (NHR) signaling pathway is an attractive candidate for a signaling pathway mediating *daf-16*/FOXO cell-nonautonomy. *daf-12*/NHR signaling is known to play a key role in coordinating a variety of systemic effects, including dauer formation, aging, and developmental timing [28]. The Rieske oxygenase DAF-36 and the cytochrome P450 DAF-9 are necessary for the production of dafachronic acid, which is a ligand for the nuclear hormone receptor DAF-12 [29–33]. Ligand-bound DAF-12 promotes dauer bypass and reproductive development, and ligand-free DAF-12, together with its co-repressor DIN-1/SHARP, promotes dauer formation [34,35].

Another potential candidate for a signaling pathway mediating the effects of FOXO cell-nonautonomy is the Transforming Growth Factor-β (TGF-β) Sma/Mab pathway. This pathway was first identified by mutations causing small body size and male tail abnormalities [36–38]. Core pathway components include the TGF-β ligand DBL-1, the TGF-β receptor subunits DAF-4 and SMA-6, the SMADs SMA-2, SMA-3, and SMA-4, and the SMAD co-factor SMA-9 [38–43]. The *dbl-1*/TGF-β pathway has also been shown to regulate reproductive aging, aversive olfactory learning, and mesodermal patterning [44–46].

Here we show that *daf-16*/FOXO promotes L1 arrest cell-nonautonomously. mRNA-seq reveals that *daf-16*/FOXO has overlapping but distinct effects on gene expression in starved L1 larvae compared to young adults with reduced insulin/IGF signaling. Our mRNA-seq analysis identified Sma/Mab TGF-β and *daf-12*/NHR pathways as candidate mediators of FOXO cell-nonautonomy. We show that *daf-16*/FOXO promotes developmental arrest throughout the animal by inhibiting expression of Sma/Mab TGF-β and *daf-12*/NHR steroid hormone signaling pathway components. These pathways promote development in fed L1 larvae and in starved *daf-16*/FOXO mutants, but their activation does not cause the early demise of *daf-16*/FOXO mutants during starvation.

## Results

### *daf-16*/FOXO regulates L1 arrest cell-nonautonomously

**Fig 1:**
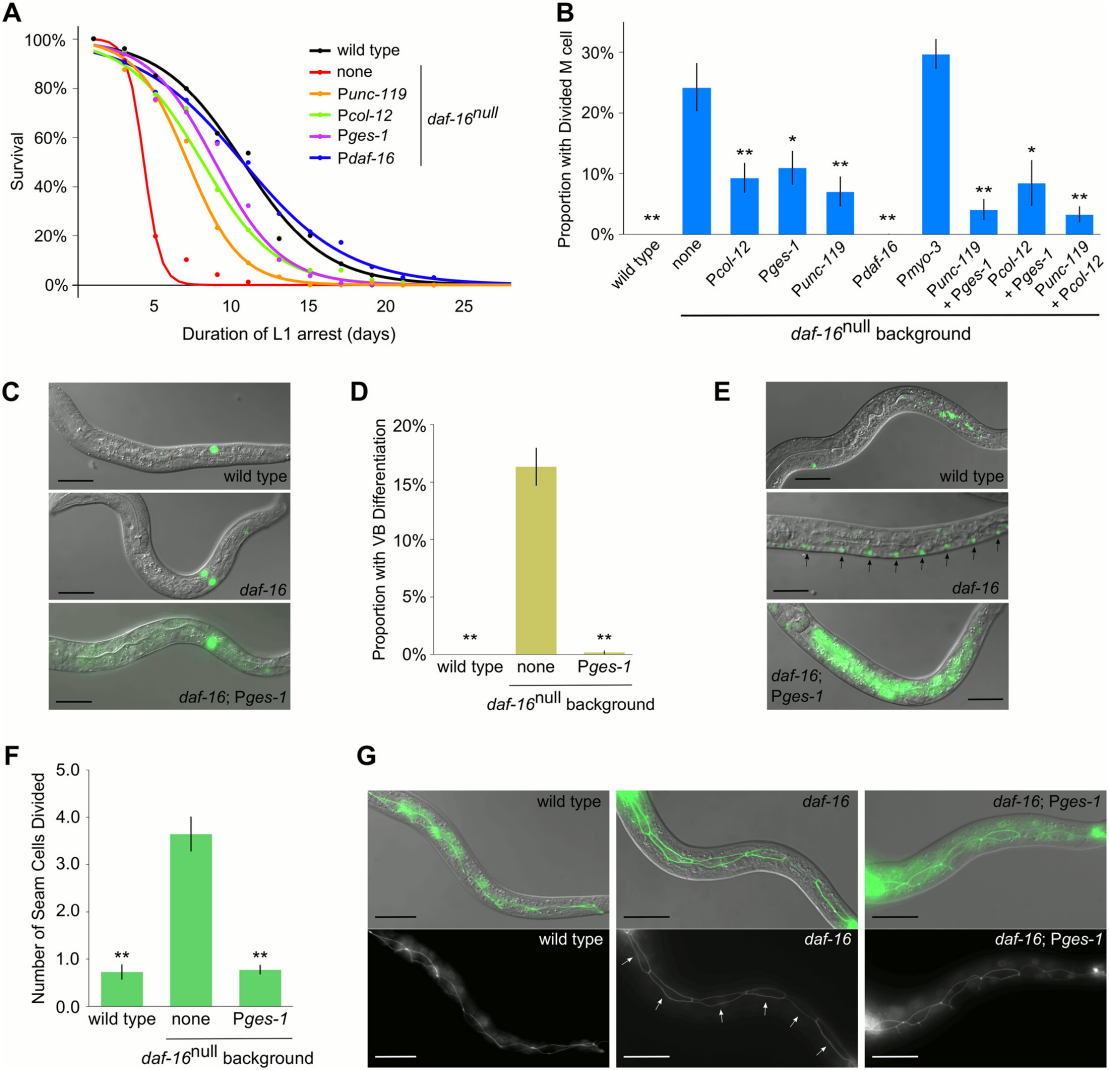
*daf-16*/FOXO regulates L1 arrest cell-nonautonomously. Tissue-specific DAF-16::GFP was assessed for rescue of *daf-16* ^*null*^ starvation phenotypes. (A) L1 starvation survival is plotted over time for a single integrated high-copy array for each promoter. A logistic regression of mean survival from at least three biological replicates is shown. See S1 Table for statistical analysis. (B) The proportion of larvae with at least one M lineage division after seven days of starvation is plotted. Combined data from three to four independent transgenic lines and at least three biological replicates is plotted for each tissue-specific rescue. See S2 Table for statistical analysis and S2 Fig for allelic breakdown. (C) Differential interference contrast (DIC) image merged with GFP fluorescence image of wild type, *daf-16* ^*null*^, and a representative tissue-specific *daf-16* rescue with the *Phlh-*8::GFP reporter after seven days of starvation, showing one, two and one M lineage cells, respectively. Scale bar: 20μm (D) The proportion of larvae with VB motor neuron differentiation after seven days of starvation is plotted for three biological replicates. (E) DIC merged with GFP of wild type, *daf-16* ^*null*^, and a representative tissue-specific *daf-16* rescue with the P*del-1*::GFP reporter after seven days of starvation. Arrows point to pairs of differentiated VB motor neurons only detectable in *daf-16* ^*null*^. Scale bar: 20μm (F) The average number of seam cell divisions, out of six possible, after three days of starvation is plotted for three biological replicates. (G) DIC merged with GFP and GFP alone of wild type, *daf-16* ^*null*^, and a representative tissue-specific *daf-16* rescue with the AJM-1::GFP reporter after three days of starvation. Arrows point to the posterior daughters of divided seam cells in *daf-16* ^*null*^ that have elongated to restore contact with one another after their anterior sisters fused with the hypodermal syncytium (see S3 Fig for the progression of seam cell development). Scale bar: 20μm (B, D, F) Error bars reflect standard error of the mean (SEM). **p<0.01, *p<0.05; unpaired t-test against *daf-16* ^null^. (C, E, G) DAF-16::GFP expression is visible in the intestine of animals carrying the integrated P*ges-1* rescue transgene.

Loss of *daf-16*/FOXO reduces starvation survival during L1 arrest [3,47]. To determine if *daf-16*/FOXO expression in a specific tissue is sufficient to rescue starvation survival, tissue-specific promoters were used to express GFP-tagged DAF-16 in a *daf-16* ^*null*^ background [18,48]. Visible DAF-16::GFP expression patterns were restricted to the tissues targeted by each promoter. Expression from the native *daf-16* promoter resulted in complete rescue of the starvation survival defect while expression from neuronal (P*unc-119*), intestinal (P*ges-1*), and epidermal (P*col-12*) promoters resulted in significant partial rescue (Fig 1A, S1 Table). Additional transgenic lines with the same promoters provided comparable results (S1 Fig). These data show that DAF-16::GFP expression in various individual tissues is sufficient to affect a whole-animal phenotype. Though it is plausible that individual tissues could have an additive effect on survival, we were unable to detect differences between single and double promoter rescues (S1 Fig). These results suggest that *daf-16* functions cell-nonautonomously during L1 arrest as it does for adult lifespan [16,18–20].

*daf-16* mutants inappropriately initiate somatic postembryonic development during starvation [3]. To determine if *daf-16*/FOXO regulates developmental arrest cell-nonautonomously, a variety of markers were examined in tissue-specific DAF-16::GFP rescue strains. The M cell is a mesoblast that undergoes a series of divisions to produce 18 cells during L1 development [49]. The M cell lineage was visualized using a P*hlh-8*::GFP reporter [50]. The M cell of wild-type larvae did not divide during L1 arrest, though a significant proportion of *daf-16* ^*null*^ larvae had at least one M lineage division (Fig 1B). Expression of DAF-16::GFP from the native promoter (P*daf-16*) resulted in complete rescue while expression from the intestinal (P*ges-1*), neuronal (P*unc-119*) and epidermal (P*col-12*) promoters resulted in significant partial rescue of the *daf-16* ^*null*^ phenotype. The muscle promoter (P*myo-3*) had no effect. Pairs of tissue-specific promoters also provided significant rescue but were not significantly different from rescue with single promoters, failing to provide evidence of additive effects (S2 Table). There appeared to be variation between independent transgenic lines for each promoter and promoter pair, suggesting allele-specific effects can confound effects of promoter/site of expression (S2 Fig). Rescue of the M cell lineage division defect by expression of DAF-16::GFP in other cells implies that *daf-16*/FOXO can function cell-nonautonomously during L1 arrest.

We investigated cells from different lineages with different developmental fates and behaviors to determine if the cell-nonautonomous effect of *daf-16*/FOXO extends to development of the entire animal. The intestinal rescue line had the strongest effect of the integrated lines tested, so we used it for these assays. P cells migrate ventrally and divide, and some of their descendants differentiate into the VB motor neurons near the L1 molt [49]. The P*del-1*::GFP reporter is expressed specifically in differentiated VB motor neurons in late L1 larvae [51]. Expression was not detectable in wild type during L1 arrest, but the reporter was active in a significant proportion of *daf-16* ^*null*^ larvae, reflecting inappropriate VB differentiation (Fig 1D and 1E). Expression of DAF-16::GFP from the intestinal promoter suppressed inappropriate differentiation (Fig 1D and 1E). The lateral epidermal seam cells divide about five hours after hatching in fed larvae [49,52]. AJM-1::GFP marks the adherens junctions of the seam cells, enabling progression of seam cell development to be visualized during L1 development (S3 Fig) AJM-1::GFP was used to determine if seam cells v1–6 divide in starved larvae [53]. *daf-16* ^*null*^ larvae had significantly more divisions than wild type when starved (Fig 1F and 1G), as expected [3]. Intestinal expression of DAF-16::GFP suppressed the division of seam cells in otherwise *daf-16* ^*null*^ animals (Fig 1F and 1G). These results show that *daf-16* can function cell-nonautonomously to promote developmental arrest in a variety of tissues throughout the body, consistent with systemic regulation.

### *daf-16*/FOXO cell-nonautonomy is unlikely to be mediated through insulin/IGF signaling

Cell-nonautonomous function of *daf-16*/FOXO suggests that it regulates at least one additional signaling pathway. FOXO-to-FOXO signaling cannot account for *daf-16*/FOXO cell-nonautonomy, since *daf-16*/FOXO is not available as an effector of insulin/IGF signaling in the affected cells. That is, when expressing *daf-16*/FOXO in specific tissues in a *daf-16* ^*null*^ background *daf-16* is absent from the cells being assayed for division. However, feedback regulation of insulin/IGF signaling could nonetheless be responsible if an alternative effector were involved (i.e. "FOXO-to-effector X" signaling). Double mutant analysis of *daf-2*/InsR and *daf-16*/FOXO showed that *daf-2*/InsR was not epistatic to *daf-16*/FOXO for M cell division (S4 Fig), arguing against this possibility. Insulin-like peptides *ins-4*, *ins-6*, and *daf-28* function redundantly to promote L1 development [54]. Simultaneous disruption of *ins-4*, *ins-5*, *ins-6*, and *daf-28* along with *daf-16*/FOXO showed that these insulin-like peptides were also not epistatic to *daf-16*/FOXO (S4 Fig). The *daf-2* allele used is not null (null is lethal) and other insulin-like peptides could be involved, but these results suggest that the cell-nonautonomous effects of *daf-16*/FOXO on M cell division are not mediated through insulin/IGF signaling.

### mRNA-seq analysis of *daf-16*/FOXO during L1 arrest

**Fig 2:**
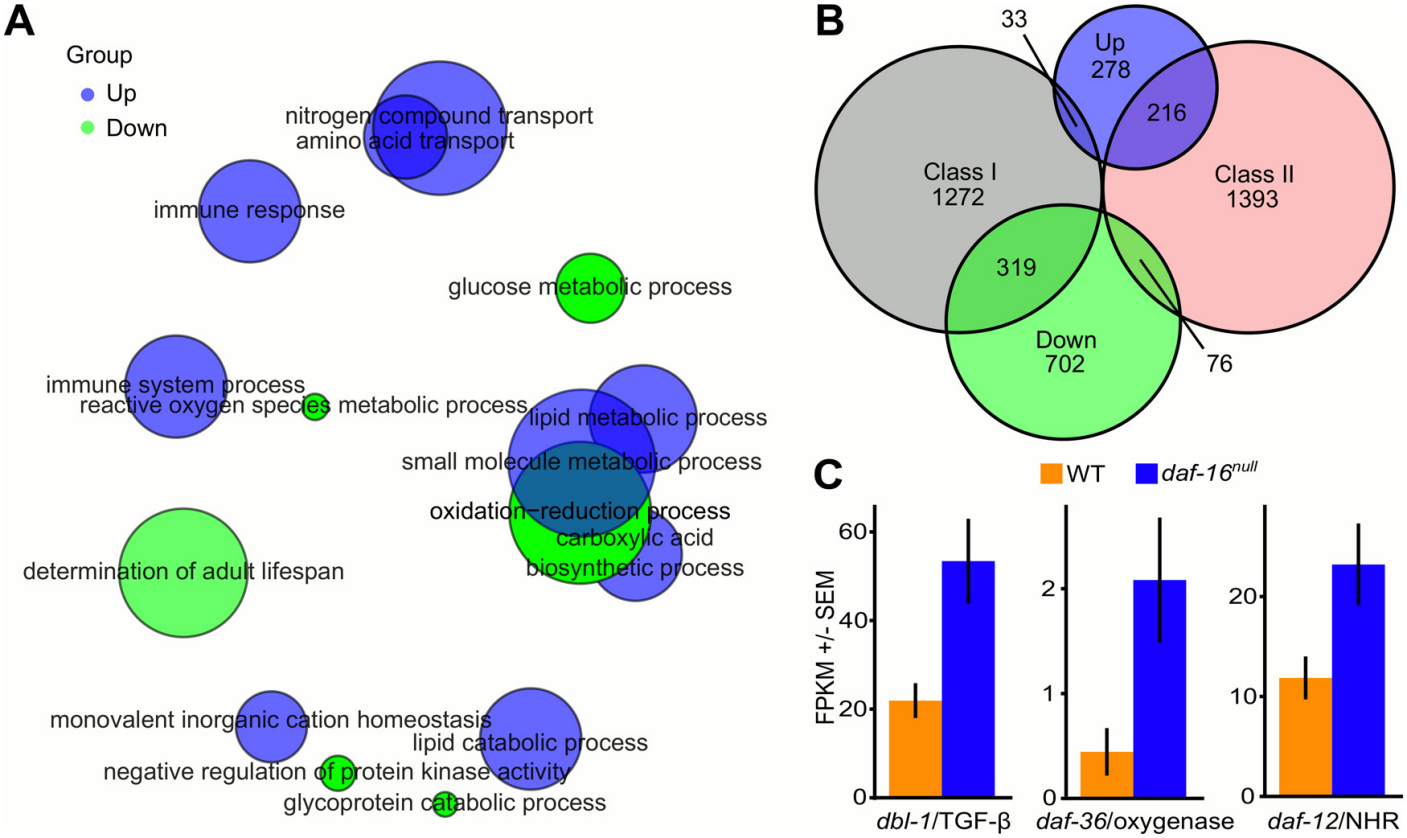
mRNA-seq analysis reveals widespread transcriptional effects of *daf-16*/FOXO during L1 arrest, including repression of *dbl-1*/TGF-β and *daf-12*/NHR pathway components. (A) GO term enrichments for "Biological Process" are plotted in semantic space by REVIGO. Bubble size corresponds to the number of genes for each term. Blue bubbles are for terms enriched among genes up-regulated in the mutant and green bubbles are for terms enriched among down-regulated genes. (B) Overlap between genes expressed higher and lower in *daf-16* ^*null*^ ("Up" and "Down", respectively) with those classified as Class I or Class II is plotted [9]. The universal set of genes considered includes only those analyzed in both studies (see S1 Dataset). (C) Average transcript abundance +/- SEM is plotted for candidate signaling molecules in wild type and *daf-16* ^*null*^ during L1 arrest. FPKM stands for fragments per kilobase per million.

We used mRNA-seq for expression analysis during L1 arrest to identify candidate signaling molecules regulated by *daf-16*/FOXO activity. A variety of studies have investigated the effects of *daf-16* on gene expression in the context of aging. These studies used fed *daf-2*/InsR adults as a background within which the effect of *daf-16* mutation was examined. We suspected that the effects of *daf-16*/FOXO on gene expression during L1 arrest were sufficiently different from its effects in young adult *daf-2*/InsR mutants to warrant analysis of L1 arrest in a wild-type background. mRNA-seq analysis of wild type and *daf-16* ^*null*^ worms on the first day of L1 starvation identified 1,353 genes with reduced expression and 558 genes with increased expression in the mutant with a false-discovery rate (FDR) of 5% (S1 Dataset). This analysis revealed widespread effects of DAF-16/FOXO on gene expression in starved L1 larvae, consistent with the phenotypic consequences of its disruption. Gene Ontology (GO) term enrichment analysis revealed a variety of effects on metabolic and immune system gene expression, with distinct patterns of enrichment for genes up- and down-regulated in the mutant and no overlapping terms between the two gene sets (Fig 2A, S1 Dataset). Notably, the term "determination of adult lifespan" was prominently enriched among genes down-regulated in the mutant, consistent with the known role of *daf-16*/FOXO in promoting lifespan. These results suggest that we effectively captured the effects of *daf-16*/FOXO on gene expression underlying the phenotypic consequences of its disruption during L1 arrest.

Our mRNA-seq analysis revealed similar but different effects of *daf-16*/FOXO on gene expression during L1 arrest compared to other contexts. A meta-analysis of multiple different genome-wide analyses of *daf-16*/FOXO-dependent gene expression in young adult animals with a *daf-2*/InsR mutant background has been published [9]. We compared the genes identified in our analysis to the "Class I" and "Class II" genes defined in the meta-analysis (Class I genes have reduced expression in the *daf-16*/FOXO mutant, as if activated by *daf-16*, and Class II genes have increased expression, as if repressed) to validate our mRNA-seq results. We found significant overlap between Class I genes and those with decreased expression in the *daf-16*/FOXO mutant during L1 arrest ("Down"; Fig 2B; hypergeometric p-value = 2.8e-88). We also found significant overlap between Class II genes and those with increased expression in the *daf-16*/FOXO mutant during L1 arrest ("Up"; hypergeometric p-value = 5.3e-87). These observations corroborate our results in that there is significant overlap with the effects of activating *daf-16*/FOXO via starvation in L1 larvae compared to activating *daf-16*/FOXO with reduction of *daf-2*/InsR function in adults. However, because of differences in experimental design, this comparison also allowed us to determine the extent to which the genes affected by *daf-16*/FOXO activity depends on stage (L1 larvae vs. young adults) and condition (wild type vs. *daf-2* mutant and starved vs. fed). Despite the significance of the overlap, the majority of the Up and Down genes we identified were not identified as Class II or Class I genes, respectively (Fig 2B). This result shows that *daf-16*/FOXO-dependent effects on gene expression are sensitive to stage and/or condition.

We examined the Up and Down genes for candidate signaling molecules that could mediate cell-nonautonomous effects of *daf-16*/FOXO. *dbl-1*/TGF-β expression was increased 2.1-fold in the mutant (FDR = 0.8%; Fig 2C; S1 Dataset), though other components of the Sma/Mab pathway were not significantly affected. In addition, expression of the Rieske oxygenase *daf-36* was increased 3.6-fold (FDR = 3%). DAF-36 is necessary to produce the ligand for DAF-12/NHR, and *daf-12*/NHR expression was increased 1.7-fold (FDR = 11%), though only marginally significant. Other components of the *daf-12*/NHR pathway were not significantly affected. The NanoString nCounter platform was used to measure transcript abundance and validate the mRNA-seq results for *dbl-1*, *daf-36*, and *daf-12*. *dbl-1* and *daf-12* were expressed significantly higher in *daf-16* ^*null*^ mutants (p<0.005; S5 Fig). Although *daf-36* showed a marginally significant increase in expression (p = 0.04), it was below the limits of reliable detection for this assay so we performed qRT-PCR [55]. qRT-PCR of *daf-36* showed an increase of 1.7-fold in the mutant (SEM = 0.32; p = 0.01). These results suggest *daf-16*/FOXO activity during L1 arrest leads to repression of *dbl-1*/TGF-β, *daf-36* and *daf-12*/NHR.

Given the effect of *daf-16/*FOXO on the *dbl-1*/TGF-β and *daf-36*/oxygenase pathways, we hypothesized that the transcription factors SMA-9/co-SMAD, the downstream effector of *dbl-1* signaling, and DAF-12/NHR may contribute to the differential expression of genes in the *daf-16* ^*null*^ mutant. We performed transcription factor binding motif enrichment analysis for DAF-16 (DBE motif; positive-control), SMA-9/co-SMAD (DBD-1 and DBD-2 motifs of the homolog Schnurri), and DAF-12/NHR (M-2 motif) [56–58]. We also performed motif enrichment analysis for PQM-1 (DAE motif), a transcription factor known to be associated with both Class I and Class II genes but not implicated in young larvae [9]. As expected, we found enrichment for the DAF-16 motif (DBE) in the Down genes (p = 9.4x10^-6^). We also found enrichment for the PQM-1 motif (DAE) in both Up and Down genes (p = 3.1x10^-47^ and p = 1.0x10^-7^, respectively), suggesting a functional role of *pqm-1* in L1 larvae. We failed to detect enrichment of the DAF-12/NHR motif in either set of genes. However, DAF-12/NHR may act on a small but functionally important set of genes, and DAF-16/FOXO is likely to regulate other genes that affect expression, both of which could limit our ability to detect enrichment of the DAF-12/NHR motif. We found enrichment of the SMA-9/co-SMAD motif (DBD-1) in the Down genes (p = 0.023). SMA-9/SMAD has been shown to act as both a transcriptional activator and repressor [59], and this result suggests it actively represses a significant number of the Down genes. This result suggests that a subset of DAF-16-regulated genes are direct targets of the *dbl-1*/TGF-β signaling pathway.

### TGF-β and steroid hormone signaling are epistatic to daf-16/FOXO for developmental arrest

**Fig 3:**
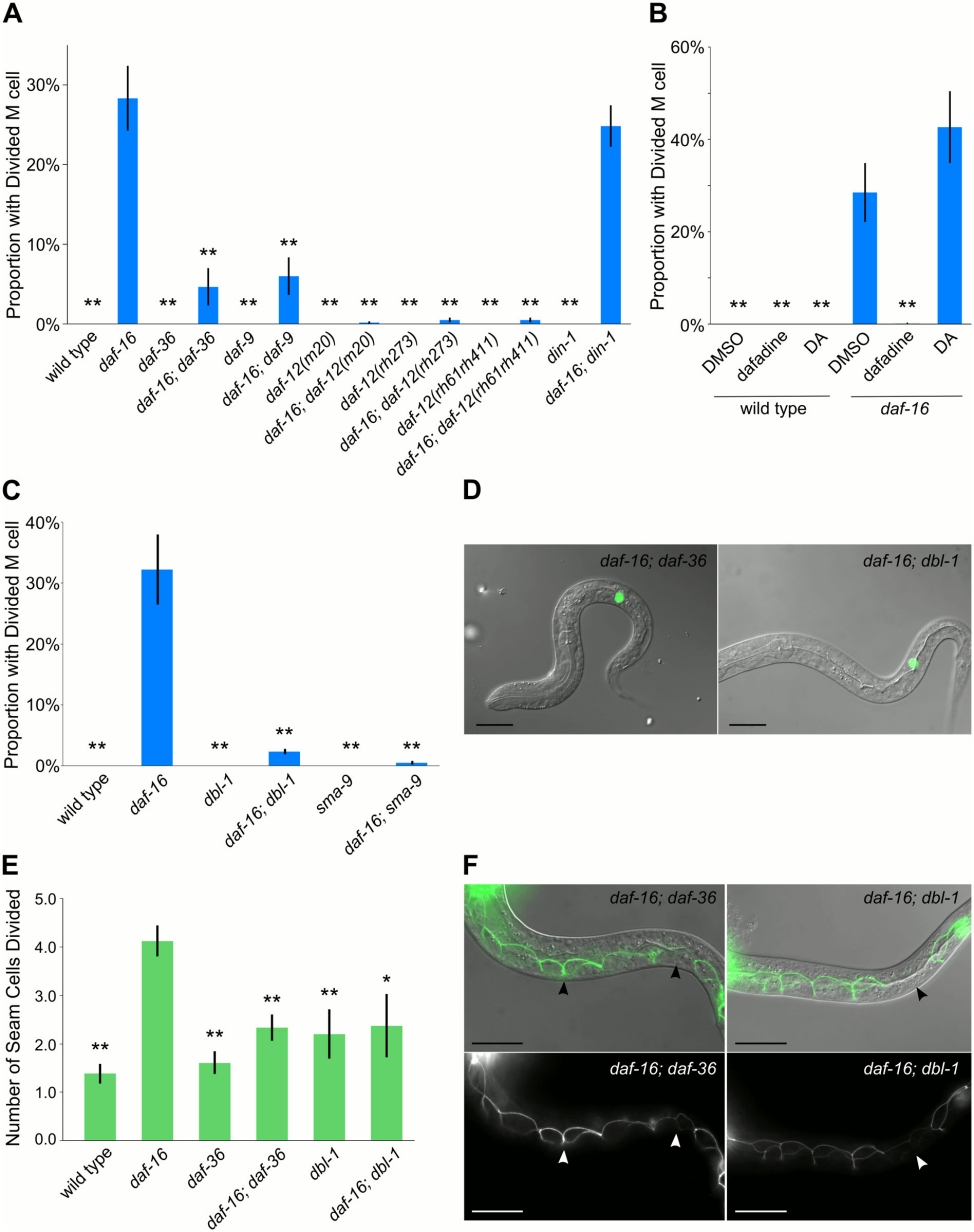
*daf-12*/NHR and *dbl-1*/TGF-β signaling pathways mediate cell-nonautonomous effects of *daf-16* on developmental arrest. (A-C) The proportion of larvae with at least one M lineage division after seven days of starvation is plotted for various genotypes and treatments. (B) Dimethyl sulfoxide (DMSO; solvent), dafachronic acid (DA). (D) DIC merged with GFP of *daf-16; daf-36* and *daf-16; dbl-1* with the *Phlh-*8::GFP reporter after seven days of starvation. Only one M lineage cell is visible in each case; compare Fig 1C. Scale bar: 20μm (E) The average number of divided seam cells after three days of starvation is plotted. (A-C, E) Error bars reflect SEM of three-seven biological replicates. *p<0.05; **p≤0.01; unpaired t-test against *daf-16*. (F) DIC merged with GFP and GFP alone of *daf-16; daf-36* and *daf-16; dbl-1* with AJM-1::GFP after three days of starvation. Arrowheads point to seam cells that have recently divided. Compare Figs 1G and S3. Scale bar: 20μm.

We used genetic epistasis analysis to determine if the effects of *daf-16*/FOXO on gene expression identified by mRNA-seq are functionally significant *in vivo*. Specifically, we hypothesized that up-regulation of *daf-12*/NHR signaling in the *daf-16* ^*null*^ mutant contributes to its arrest-defective phenotype. Components of the pathway *daf-36*/oxygenase, *daf-9*/CYP450, and *daf-12*/NHR were epistatic to *daf-16*/FOXO with respect to M cell division (Fig 3A and 3D). *daf-12(m20)* is a null allele for all but the B isoform, which is a short isoform containing the ligand-binding domain (LBD) but not the DNA-binding domain (DBD). *rh61rh411* is a null allele for all *daf-12* isoforms. *daf-12(rh273)* contains a substitution in the LBD, which is hypothesized to interrupt ligand binding [34]. All three alleles suppressed the *daf-16* ^*null*^ arrest-defective phenotype. *din-1*/SHARP collaborates with ligand-free *daf-12* as a co-repressor in promoting dauer formation [35]. *din-1* was dispensable for the *daf-16* ^*null*^ arrest-defective phenotype (Fig 3A), suggesting that ligand-bound *daf-12*/NHR promotes M cell division and that it is normally inhibited by *daf-16*/FOXO activity during starvation.

Pharmacological manipulation of the *daf-12*/NHR pathway corroborated genetic analysis. Dafadine is a small molecule inhibitor of *daf-9* [60]. Wild-type and *daf-16* ^*null*^ worms were exposed to dafadine or dafachronic acid during starvation. Dafachronic acid did not cause an arrest-defective phenotype, suggesting that the steroid hormone pathway is not sufficient to promote development. Dafadine suppressed M cell lineage divisions in *daf-16* ^*null*^ worms (Fig 3B), phenocopying *daf-9(m540)*. These results further support the conclusion that *daf-16*/FOXO activity inhibits *daf-12*/NHR signaling during L1 arrest, and that this inhibition is functionally significant.

Given the mRNA-seq results, we also hypothesized that up-regulation of *dbl-1*/TGF-β signaling in the *daf-16* ^*null*^ mutant contributes to its arrest-defective phenotype. Mutations affecting *dbl-1*/TGF-β and its downstream effector *sma-9*/co-SMAD suppressed the *daf-16* ^*null*^ arrest-defective phenotype (Fig 3C and 3D). These results suggest that *daf-16*/FOXO also inhibits *dbl-1*/TGF-β signaling during L1 arrest, and that this inhibition has physiological consequences.

*daf-12*/NHR and *dbl-1*/TGF-β signaling were also required for inappropriate seam cell divisions in starved *daf-16* ^*null*^ worms. Both *daf-36* and *dbl-1*/TGF-β were epistatic to *daf-16*/FOXO for seam cell division (Fig 3E and 3F). Overall, these data suggest that *daf-16*/FOXO activity leads to repression of both Sma/Mab TGF-β and *daf-12*/NHR signaling during starvation to promote developmental arrest.

### TGF-β and steroid hormone signaling promote early larval development

**Fig 4:**
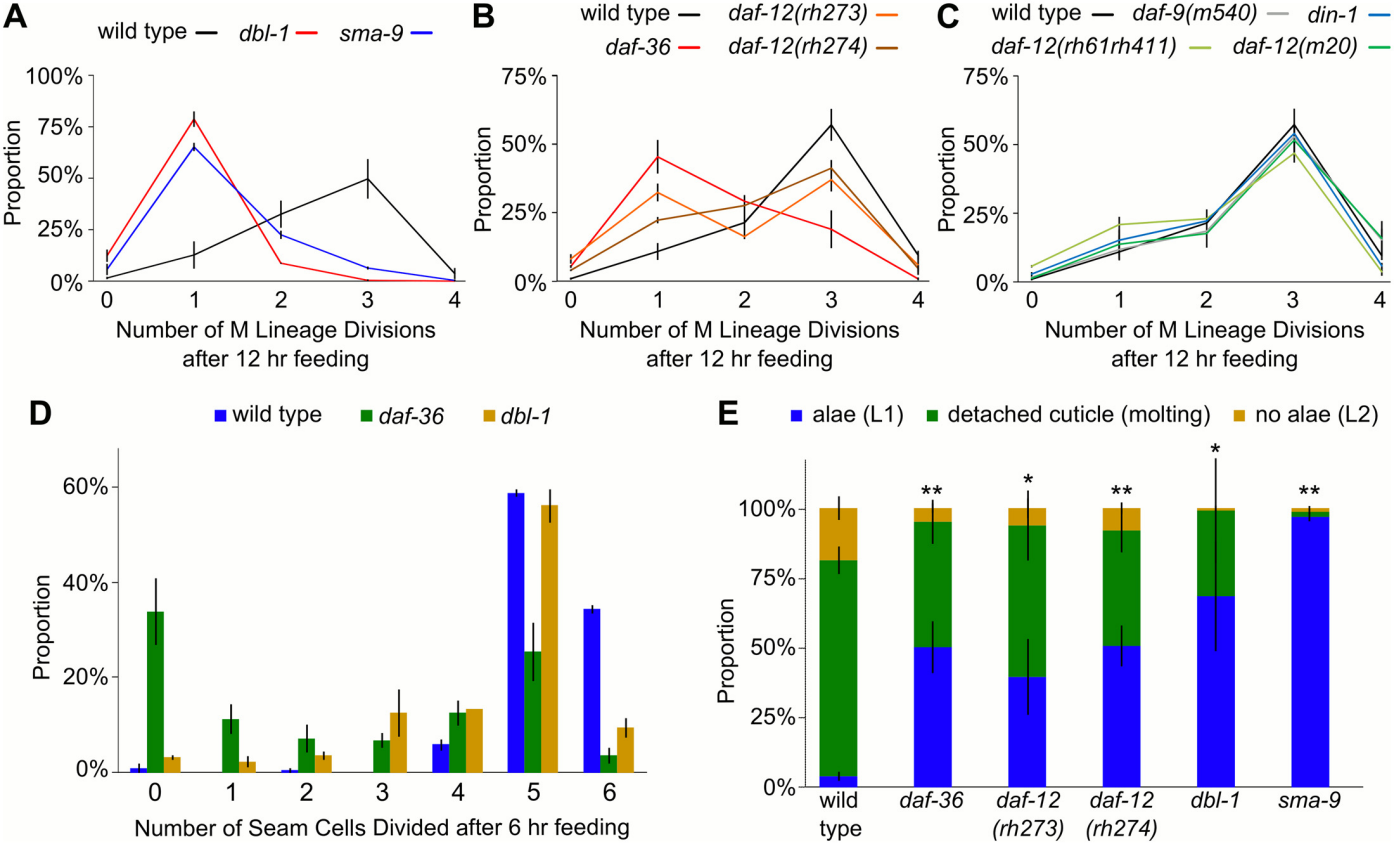
*daf-12*/NHR and *dbl-1*/TGF-β signaling pathways promote L1 development in fed larvae. (A-C) The proportion of larvae with 0–4 M lineage divisions after 12 hours recovery from L1 arrest at 20°C is plotted. Error bars reflect SEM of at least three biological replicates. (A) *dbl-1* and *sma-9* mutants have a lower average number of divisions than wild type (p<0.01; unpaired t-test). (B) *daf-36*, *daf-12(rh273)*, and *daf-12(rh274)* mutants have fewer average divisions than wild type (p<0.05; unpaired t-test). (C) *daf-9* ^*hypmorph*^, *daf-12* ^*null*^, and *din-1* mutants are not significantly different from wild type. (D) The proportion of larvae with 0–6 seam cell divisions after six hours recovery from L1 arrest at 20°C is shown. *dbl-1* and *daf-36* mutants have fewer average seam cell divisions than wild type (p<0.01; unpaired t-test). (E) Larvae scored for molting by observation of the presence or absence of L1-specific alae or a detached cuticle after 18 hours recovery from L1 arrest at 20°C is shown. *dbl-1* and *daf-12* pathway mutants show delayed molting compared to wild type (*p<0.05, **p<0.01; unpaired t-test).

We hypothesized that *daf-12*/NHR and *dbl-1*/TGF-β signaling promote L1 development in fed larvae, since they are required for the *daf-16* ^*null*^ arrest-defective phenotype, though such early larval function has not been shown. We used P*hlh-8*::GFP, AJM-1::GFP and molting to monitor the rate of L1 development in fed larvae with mutations in these pathways. *dbl-1*/TGF-β and *sma-9*/co-SMAD mutants clearly had delayed M cell lineage divisions (Fig 4A), consistent with our hypothesis. The *daf-36* mutant also had significantly fewer M cell divisions on average than wild type (Fig 4B), suggesting that ligand-bound DAF-12 promotes L1 development. *daf-12(rh273)* and *daf-12(rh274)*, which have a constitutive dauer-formation phenotype due to mutations in the LBD, also caused developmental delay (Fig 4B). These results suggest that ligand-free DAF-12 represses L1 development, analogous to its repression of reproductive development during dauer formation. However, the null alleles of *daf-12* had no effect (Fig 4C), which we believe is due to the opposing effects of ligand-bound and -free forms of DAF-12. This interpretation is supported by the fact that these same alleles do not cause a constitutive dauer-formation phenotype [34]. The null allele for *din-1*, the co-repressor for ligand-free DAF-12, also had no effect on M cell lineage division rate, as expected based on our model and its role in regulation of dauer formation. *daf-9(m540)* is a suspected hypomorph [30], and residual activity appeared to be sufficient for normal developmental rate (Fig 4C). These results support the conclusion that *daf-12*/NHR and *dbl-1*/TGF-β signaling promote M cell lineage division in fed L1 larvae.

We assessed seam cell divisions and molting progression in fed larvae to broaden our developmental analysis, examining developmental events that occur before and after M cell division, respectively. *daf-36* and *dbl-1*/TGF-β mutants had significantly fewer seam cell divisions than wild type (Fig 4D). L1 larvae have a stage-specific cuticular ridge (alae) on their lateral midline that is absent from L2 larvae. The presence or absence of alae was scored, along with detached cuticles, to identify L1 or L2 larvae, and larvae undergoing the L1 molt, respectively. The L1 molt was significantly delayed in mutants of the *daf-36* and *dbl-1* pathways compared to wild type (Fig 4E). These data further support the conclusion that Sma/Mab TGF-β and *daf-12*/NHR signaling promote L1 development in fed larvae.

### TGF-β and steroid hormone signaling do not affect starvation survival

**Fig 5:**
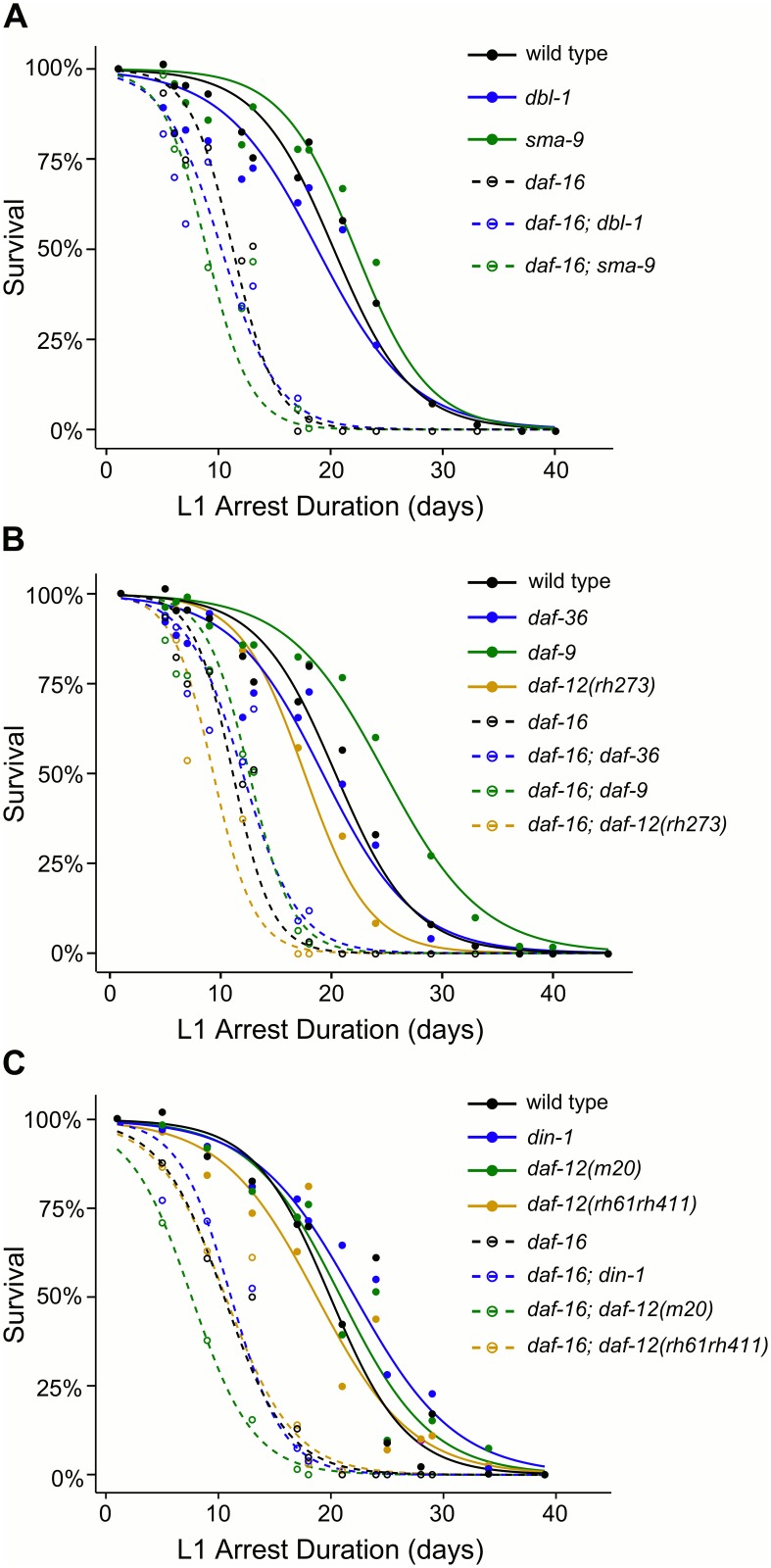
*daf-12*/NHR and *dbl-1*/TGF-β signaling do not modify effects of *daf-16* ^*null*^ on starvation resistance. (A-C) L1 starvation survival is plotted over time. A logistic regression of mean survival from at least three biological replicates is shown. All strains containing a *daf-16* mutation have significantly lower survival than wild type and are not significantly different from *daf-16*, and none of the single mutants are significantly different than wild type (see S3 Table for statistical analysis). (A-B) All strains included the P*hlh-8*::GFP reporter in the background.

Does initiation of post-embryonic development in *daf-16* ^*null*^ cause reduced starvation survival? *dbl-1*/TGF-β and *sma-9*/co-SMAD were not epistatic to *daf-16*/FOXO for starvation survival (Fig 5A; S3 Table). Mutations affecting steroid hormone pathway components *daf-36*, *daf-9*, and *daf-12*/NHR also did not affect starvation survival in a wild-type or *daf-16* ^*null*^ background (Fig 5B; S3 Table). *din-1*/SHARP and *daf-12*/NHR null alleles also had no effect on starvation survival in either background (Fig 5C; S3 Table). Together these results suggest that *dbl-1*/TGF-β and *daf-12*/NHR signaling do not affect starvation survival, implying that development during starvation is not the cause of *daf-16* ^*null*^ starvation sensitivity.

## Discussion

**Fig 6:**
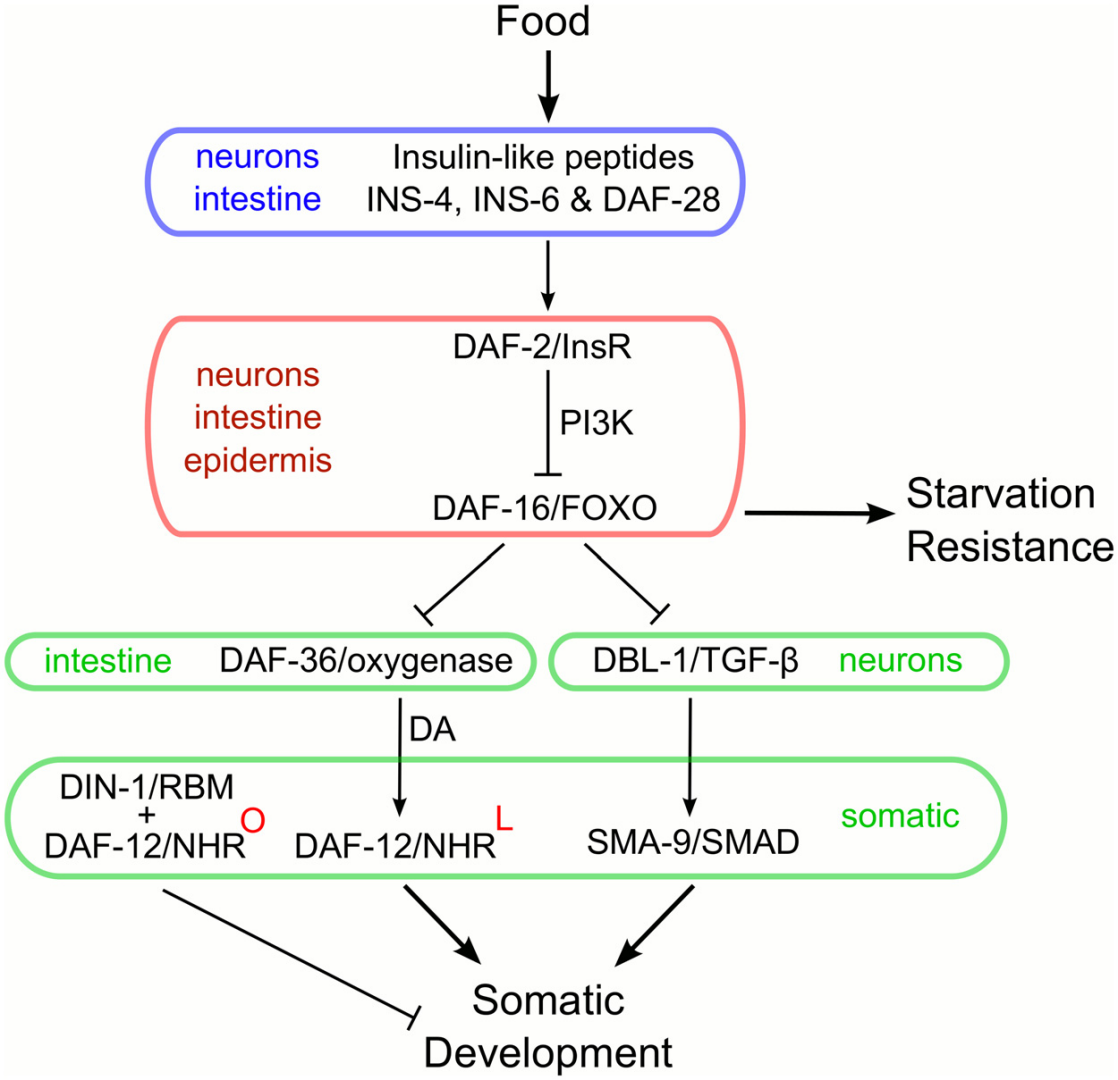
*daf-16*/FOXO promotes developmental arrest by inhibiting *daf-12*/NHR and *dbl-1*/TGF-β signaling. In our model, the insulin-like peptides INS-4, INS-6, DAF-28 and possibly others are secreted from neurons and the intestine in L1 larvae in response to feeding. These peptides function as agonists of DAF-2/InsR, leading to the cytoplasmic localization and repression of DAF-16/FOXO via PI3K signaling in the intestine, neurons and epidermis. With DAF-16/FOXO repressed, *dbl-1* Sma/Mab TGF-β and *daf-12* steroid hormone pathways are activated to promote early larval development. DAF-12/NHR signaling is activated via production of its steroid ligand, dafachronic acid (ligand-bound DAF-12 is indicated by a red "L" in superscript). During starvation insulin/IGF signaling is dramatically reduced, allowing DAF-16/FOXO to enter the nucleus where it directly or indirectly represses *dbl-1* Sma/Mab TGF-β and *daf-12* steroid hormone signaling. Inhibition of these pathways promotes developmental arrest cell-nonautonomously. In the absence of dafachronic acid, DAF-12/NHR along with its co-repressor DIN-1/SHARP antagonizes development (ligand-free DAF-12 is indicated by a red "O" in superscript). Notably, repression of primordial germ cell division involves insulin/IGF and PI3K signaling but does not depend on *daf-16*/FOXO. These downstream signaling pathways do not affect starvation survival, indicating that DAF-16/FOXO acts through different target genes to promote starvation resistance. Colored boxes reflect anatomical sites of action, with color corresponding to levels in the signaling network as opposed to sites.

This work shows that *daf-16*/FOXO functions cell-nonautonomously to regulate *C*. *elegans* L1 arrest. A recent study corroborates this finding, highlighting the need to determine the molecular basis of cell-nonautonomous function [61]. The *dbl-1*/TGF-β and *daf-12*/NHR signaling pathways have not been reported to affect early larval development, but our results reveal that these pathways promote L1 development in fed larvae. mRNA expression analysis together with genetic epistasis analysis suggest that *daf-16*/FOXO inhibits these pathways to promote developmental arrest. Taken together, this work shows that *daf-16*/FOXO promotes L1 arrest by inhibiting pathways that promote development (Fig 6).

Our results suggest that *daf-16*/FOXO can function in the intestine, nervous system or epidermis to promote starvation survival and developmental arrest. We analyzed four independent transgenic lines per tissue-specific rescue construct. Apparent allele-specific effects confounded our ability to distinguish tissue-specific effects on rescue. Variation in promoter strength may also obscure tissue-specific effects. These technical complications limit our ability to conclude whether *daf-16*/FOXO activity in one tissue is functionally more important than another. Though we could not statistically distinguish the effects of rescue in intestine, nervous system or epidermis, we clearly found that muscle rescue had no effect on starvation survival or developmental arrest. Expression in the intestine, nervous system or epidermis was sufficient to partially rescue *daf-16* ^*null*^, but rescue was not complete, though it was complete when the native *daf-16* promoter was used. We considered that additive effects of multiple tissues might explain this finding, but we did not observe a significant change in penetrance when pairs of promoters were used for rescue. Our starvation survival assay may have lacked sufficient dynamic range to observe this. Alternatively, and particularly with respect to developmental arrest, different components of a single pathway may be regulated by DAF-16/FOXO in different tissues such that regulation in any single tissue is sufficient to disrupt the pathway. Consistent with this interpretation, *dbl-1*/TGF-β is expressed in neurons while its receptor and downstream SMADs are expressed broadly [40,41,43,62,63]. Likewise, *daf-36* is expressed in the intestine, *daf-9*/CYP450 is expressed in the epidermis and XXX cells, and *daf-12*/NHR is expressed broadly [29,32,34,64]. We speculate that DAF-16/FOXO functions in several of these tissues to inhibit these genes directly or indirectly.

Anatomical sites of action for the insulin/IGF signaling pathway have been characterized for regulation of dauer formation, aging, reproductive aging, developmental arrest and starvation survival. These analyses have produced largely overlapping results, implicating primarily the intestine, nervous system and epidermis. Rescue of *daf-2*/InsR or *age-1*/PI3K suggested the nervous system as a key site of action in regulation of aging, but rescue of *daf-16*/FOXO in a *daf-2*/InsR mutant background suggested the intestine [18,19]. However, because *daf-2* and *age-1* antagonize *daf-16*, these two experimental designs should not be expected to produce the same results. Nonetheless, both studies actually found some effect in each site. In addition, a subsequent study found that epidermal rescue of *daf-16*/FOXO in a *daf-2*/InsR mutant background can also rescue lifespan [20]. Curiously, *daf-16*/FOXO expression in the intestine was originally found not to affect dauer formation while neural expression did [18], but more recently it was reported that *daf-16*/FOXO expression in the intestine determines dauer formation without contribution from neurons [17]. It is unclear why there are such discrepancies among these results, though specific experimental conditions, promoters, and in some cases limited numbers of transgenic alleles could contribute. Muscle expression of *daf-16*/FOXO was not found to affect lifespan or dauer formation in any of these studies, but it does affect reproductive aging [65]. Overlapping but discordant results are also evident with respect to regulation of developmental arrest. For example, the *daf-16*/FOXO target *mir-235*, which robustly phenocopies *daf-16* ^*null*^, was found to function in the epidermis and nervous system to promote developmental arrest in starved L1 larvae [66]. In contrast, transgenic rescue of *daf-18*/PTEN, which is thought to act through *daf-16*/FOXO for arrest of somatic cells, highlighted the epidermis as the primary site of action but also showed that it can function cell-autonomously as well [61]. Likewise, rescue of *daf-16*/FOXO in the context of starvation-induced post-dauer arrest also implicated the epidermis [48]. Though we found the epidermis to be an important site of *daf-16*/FOXO action in regulation of L1 arrest, our results also suggest that the nervous system and intestine are important.

Neurons and intestine also appear to be important sites of action upstream of *daf-2*/InsR and *daf-16*/FOXO. That is, the insulin-like peptides *ins-4*, *ins-6*, and *daf-28* have been shown to be regulators of L1 arrest and dauer formation with expression in chemosensory and motor neurons as well as intestine [17,54]. As the organismal signaling network mediating nutritional control of dauer formation, aging, and developmental arrest is characterized it is increasingly clear that it is complex, being distributed over several different tissues and likely involving feedback and crosstalk at a variety of levels.

Our results reveal that *dbl-1*/TGF-β and *daf-12*/NHR signaling pathways are each required for the *daf-16* ^*null*^ arrest-defective phenotype. However, the *dbl-1*/TGF-β and *daf-12*/NHR signaling pathways do not affect starvation survival, uncoupling control of development and starvation resistance, as seen with *mir-235* [66]. This finding is also consistent with previous work showing no effect of *daf-12*/NHR on starvation survival [67]. Such uncoupling suggests that *daf-16*/FOXO acts through different target genes to promote starvation resistance and developmental arrest, and that *daf-16*/FOXO mutants do not die rapidly during starvation as a result of inappropriate development.

It has been suggested that DAF-16 functions as a transcriptional activator [9,68,69], implying that repression of *dbl-1*/TGF-β and *daf-12*/NHR signaling is indirect. Our mRNA-seq analysis presumably captures direct and indirect effects, but it identified 2.4 times more genes with decreased expression in *daf-16* ^*null*^ than with increased expression, consistent with *daf-16*/FOXO having more effect as an activator than a repressor. However, DAF-16/FOXO directly binds the promoter of *daf-12*/NHR as well as components of the *dbl-1*/TGF-β pathway, including the SMADs *sma-2* and *sma-3* and *sma-9*/co-SMAD, based on DamID analysis in adults [69]. Likewise, modENCODE data for DAF-16 ChIP-seq in L3-stage larvae suggests direct binding to *daf-12*/NHR and the upstream cytochrome P450 *daf-9* as well as the TGF-β Sma/Mab receptor subunits *sma-6* and *daf-4*, and *sma-2* and *sma-3* [9,70]. In *Drosophila*, dFOXO directly binds the promoter of the TGF-β ligand *dawdle* and the *daf-12*/NHR homolog dHR96 [71,72]. These observations suggest that *daf-16*/FOXO regulation could actually be direct. *daf-16*/FOXO activates the microRNA *mir-235* during L1 arrest [66], which could function as an intermediate if regulation is indirect. However, *mir-235* is not expressed in and does not function in the intestine, a site of *daf-16*/FOXO action based on our results. DAF-16/FOXO antagonizes the transcription factor PQM-1, which promotes expression of genes up-regulated in *daf-16*/FOXO mutants [9], suggesting it too could function as an intermediate. Consistent with this possibility, we found significant enrichment of the PQM-1 binding site motif (DAE) among genes up- and down-regulated in *daf-16* ^*null*^, suggesting PQM-1 contributes to gene regulation during L1 arrest. In addition, insulin/IGF signaling and the Rag-TORC1 pathway crosstalk in regulation of L1 arrest [61], providing an additional possibility for indirect effects. It is also important to note that the changes in expression observed for *daf-36*, *daf-12*, and *dbl-1* in *daf-16* ^*null*^ were relatively small, suggesting that post-transcriptional mechanisms could also contribute to regulation.

FOXO regulates TGF-β and NHR signaling in other contexts. *daf-16*/FOXO functions upstream of *daf-36*/oxygenase, *daf-9*/CYP450, and *daf-12*/NHR in dauer formation [14,73]. *daf-9* also functions downstream of *daf-16*/FOXO in progression through post-dauer developmental checkpoints, though *daf-12*/NHR was not implicated [48]. In addition, *dbl-1*/TGF-β and insulin/IGF signaling both participate in pathogen-associated olfactory learning [10,46]. Regulation of TGF-β signaling by insulin/IGF signaling has not been reported in *C*. *elegans* or mammals, but dFOXO represses the TGF-β ligand *dawdle* in Drosophila, affecting lifespan [71]. These observations suggest that FOXO regulation of TGF-β and steroid hormone signaling pathways is broadly significant and conserved among metazoa.

## Materials and Methods

### *C*. *elegans* growth conditions and strains

Strains were maintained on agar plates containing standard nematode growth media (NGM) seeded with *E*. *coli* OP50 at 20°C. Strains containing the alleles *daf-12(rh273)* and *daf-12(rh274)* were maintained at 15°C. The wild-type strain N2 (Bristol) and the following mutants and transgenes were used: *daf-2(e979)*, *daf-2(e1370)*, *daf-16(mgDf47)*, *daf-16(mgDf50)*, *daf-16(mu86)*, *ins-4 ins-5 ins-6(hpDf761)*, *daf-28(tm2308)*, *daf-36(k114)*, *daf-9(m540)*, *daf-9(dh6)*, *daf-12(m20)*, *daf-12(rh273)*, *daf-12(rh274)*, *daf-12(rh61rh411)*, *din-1(dh127)*, *dbl-1(wk70)*, *sma-9(wk55)*, *ayIs7[Phlh-8*::*GFP]*, *syIs78[Pajm-1*::*AJM-1*::*GFP]*, *wdIs3[Pdel-1*::*GFP]*, *qyEx264[Pmyo-3*::*GFP*::*DAF-16]*, *qyIs288[Pdaf-16*::*GFP*::*DAF-16]*, *qyIs290[Pcol-12*::*GFP*::*DAF-16]*, *qyIs292[Pges-1*::*GFP*::*DAF-16]*, *qyIs294[Punc-119*::*GFP*::*DAF-16]*, *dukEx88–90[Phlh-8*::*GFP +unc-119(+) + Pcol-12*::*DAF-16*::*GFP]*, *dukEx91*,*92*,*103[Phlh-8*::*GFP +unc-119(+) + Pges-1*::*DAF-16*::*GFP]*, *dukEx93–95[Phlh-8*::*GFP +unc-119(+) + Pcol-12*::*DAF-16*::*GFP + Pges-1*::*DAF-16*::*GFP]*, *dukEx96–98[Phlh-8*::*GFP +unc-119(+) + Punc-119*::*DAF-16*::*GFP + Pges-1*::*DAF-16*::*GFP]*, *dukEx99[Phlh-8*::*GFP +unc-119(+)]*,*dukEx100–102[Phlh-8*::*GFP +unc-119(+) + Pcol-12*::*DAF-16*::*GFP + Punc-119*::*DAF-16*::*GFP]*, *dukEx104–106[Phlh-8*::*GFP +unc-119(+) + Punc-119*::*DAF-16*::*GFP]*, *dukEx107–109[Phlh-8*::*GFP +unc-119(+) + Pdaf-16*::*DAF-16*::*GFP]*, and *dukEx110–112[Phlh-8*::*GFP +unc-119(+) + Pmyo-3*::*DAF-16*::*GFP]*. Standard genetic techniques were used to create different combinations of alleles.

### Hypochlorite treatment and L1 arrest assays

Mixed-stage cultures on 10 cm NGM plates were washed from the plates using S-basal and centrifuged. A hypochlorite solution (7:2:1 ddH_2_O, sodium hypochlorite (Sigma), 5 M KOH) was added to dissolve the animals. Worms were centrifuged after 1.5–2 minutes in the hypochlorite solution and fresh solution was added. Total time in the hypochlorite solution was 8–10 minutes. Embryos were washed three times in S-basal buffer (including 0.1% ethanol and 5 ng/μL cholesterol) before final suspension in 5 mL S-basal at a density of 1.5 worms/μL. Embryos were cultured in a 16 mm glass tube on a tissue culture roller drum at approximately 25 rpm and 21–22°C.

For the M cell division and VB motor neuron differentiation assays during starvation, the larvae were starved for 7 days before 200 larvae per replicate were examined on a slide on a compound fluorescent microscope. For the dafadine and dafachronic acid experiments, 25 μM DMSO, 25 μM dafadine, or 50 nM Δ^7^-dafachronic acid was added to the final suspension in S-basal. For the seam cell division assay during starvation, the larvae were starved for 3 days and the v1–6 cells on one side of the animal were scored for 60–70 larvae per replicate.

### mRNA-seq and associated analysis

The data for wild type (N2) was published previously (GEO accession number GSE33023; "0 hr recovery") [74], and *daf-16* ^*null*^ data are first reported here. Two biological replicates were performed. Worms were cultured, RNA was prepared and sequencing and analysis were done as described [74]. The only exceptions to this pertain to the software used to count reads aligning to genes. Briefly, liquid culture was used and total RNA was prepared with TRIzol (Invitrogen). Strain GR1307 [*daf-16(mgDf50)*] was used. mRNA was isolated by polyA-selection. Sequencing libraries were prepared using the Solid Total RNA-seq Kit using the Whole Transcriptome Protocol (Applied Biosystems). Twelve PCR cycles were used to amplify the libraries. Fifty base pair single-end reads were sequenced on the Solid 4 system according to the manufacturer's protocols in the Genome Sequencing Shared Resource at Duke University. Sequencing reads were mapped using Bowtie v0.12.7 allowing two mismatches and requiring unique alignments [75] and genome coordinates for WS210. The script htseq-count [76] was used to count reads mapping to genes defined using WS220 annotation that had been mapped to WS210 coordinates. Reads were counted using the options “-m union–s yes”. DESeq v1.20.0 was used to analyze differential gene expression [77]. Cufflinks v2.1.1 was used to determine fragments per kilobase per million (FPKM; [78]). GO term enrichments were identified using GOrilla [79]. Enrichments were plotted in semantic space using REVIGO [80]. Specific parameters used with GOrilla and REVIGO are documented in S1 Dataset. GEO accession number for the *daf-16* ^*null*^ dataset is GSE69329.

### Motif enrichment analysis

Enrichment for DBE (DAF-16) and DAE (PQM-1) [9], SMAD DBD-1/DBD-2 (SMA-9) [56] and M-2 (DAF-12) [57] motifs were calculated using the AME application of the MEME software package [58]. The primary sequences scanned were the 700 bp upstream of the most upstream translation start sites of the Up and Down genes (Fig 2B) from version WS220 of the *C*. *elegans* genome. The background control promoter sequences (N = 2350) was comprised of all genes well detected in the mRNA data (expression in both WT and DAF-16 being in the top 75% percentile) and a having a strong lack of evidence of differential expression between wild-type and the DAF-16 mutant (adjusted p-value > 0.9). For each motif the background model was set to uniform, and the remaining AME parameters were default.

### Nanostring nCounter mRNA expression analysis

NanoString expression analysis was conducted as described with the following exceptions [54]. All five strains were cultured at 15°C since *daf-2* mutants are temperature sensitive. Worms were cultured in liquid at 180 rpm and 4–5 worms/μL with 40 mg/ml HB101 as food. N2, *daf-16(mgDf47)*, and *daf-16(mgDf47); daf-2(e1370)* were bleached after 5 d culture as young gravid adults. *daf-2(e1370)* and *daf-2(e979)* were bleached at 6 d and 7 d, respectively, as young gravid adults. For each strain, embryos were divided into three flasks of S-basal at 5 worms/μL and cultured at 15°C, 20°C or 25°C at 180 rpm. Arrested L1 larvae were collected 24 hr after bleaching for 20°C and 25°C, and after 48 hr for 15°C. Larvae were washed, pelleted and flash frozen. Two or three biological replicates were included for each strain. Total RNA was prepared with TRIzol, and 3 μg was used for each hybridization. Data were first normalized by spike-in controls and then normalized by three internal control genes included as targets in the codeset (*grld-1*, *rnf-5* and T16G12.6). Internal controls were identified from genome-wide time-series analysis of fed and starved L1 larvae by virtue of moderate expression levels and invariant expression over time and between conditions [74,81].

### qRT-PCR and analysis

RNA was collected from N2 and *daf-16(mgDf50)* after 1 day of starvation in S-basal. cDNA was synthesized from 1 μg of total RNA using oligonucleotide (dT) primers and SuperScript III Reverse Transcriptase (Thermo Fisher). qPCR was performed with Brilliant II QPCR Master Mix (Agilent) according to manufacturer’s protocol. The genes T16G12.6, *rnf-5*, and *grld-1* were used as internal controls (also used as internal controls for NanoString analysis). Primers were PrimeTime qPCR primers from IDT as follows (in the order of forward, probe, reverse): *daf-36*, ATCACAGACTCATATTGCCCG, TGTCACGTACTACCCGTCCTCCAA, ACACATTTTCCAGTTTCTGCAC; T16G12.6, CACCACAGACACAAGAACACTA, AACCATACGGGACATCAGCCCTTG, CGGCCAAATTGAAGCGAATC; *rnf-5*, AACCACCACCGCAATCAT, ATGCACATTTGGTCCGCCGC, TCAACGGGAACAGACCATTC; *grld-1*, AAGCTGCAGGCGTTGTAA, TGGGAAGATGTAGAGAATGCCGCC, AAGAGCTCCGAGCAAGAATG. Three technical replicates were performed for each of three biological replicates. Standard curves were analyzed to determine reaction efficiency. *daf-36* was normalized based on the internal controls and reaction efficiencies to calculate a fold-change between N2 and *daf-16* null. An unpaired t-test was performed to determine significance.

### Development assays

Following hypochlorite treatment, cultures were synchronized by overnight passage in L1 arrest at a density of 1.5 worms/μL in 5 mL S-basal. For recovery and development, 2,000 arrested L1 larvae were plated per NGM OP50 plate and placed at 20°C for 6 hours (AJM-1 marker assay), 12 hours (M cell marker assay), or 18 hours (molting progression assay). Larvae were then washed off the plate with S-basal, centrifuged, and mounted on an agarose pad. A compound fluorescent microscope was used to score cell divisions or cuticle structure in 200 worms (M cell assay) or 75 worms (seam cell and molting progression assays) per replicate.

### Starvation survival

Animals were treated in hypochlorite solution and suspended in S-basal as described above. 100 μL aliquots were sampled on different days and placed around the edge of an OP50 lawn on NGM plates. Number of plated worms (T_p_) was counted and the plates were incubated at 20°C. After two days the number of animals that survived (T_s_) was counted. Survival was calculated as T_s_/T_p_.

The *daf-16* tissue-specific starvation survival experiments were done with an alternative protocol. Instead of S-basal, virgin S-basal (no ethanol or cholesterol) was used for both hypochlorite treatment and final suspension. Worms were suspended at 1 worm/μL. For the integrated strains, 100 μL aliquots were sampled every two days and spontaneous movement in liquid was used to score survival. For the strains with an extrachromosomal array, the culture was centrifuged after seven days and the pelleted worms were placed on NGM plates. Worms expressing GFP were then scored as alive or dead based on spontaneous movement.

### Data analysis and statistics

Data were handled in R and Excel. Graphs were plotted in the R package ggplot2 or Excel. Statistical tests were performed in R or Excel. Starvation survival analysis was performed on 50% survival times (*t*_*half*_), which were obtained by fitting survival data for each trial with the function

$$S=100-\frac{100}{1+e^{(t_{half}-t)/rate}}$$

which we have modified slightly from [82]. Goodness of fit is reported as R^2^ in S1 and S3 Tables.

## Supporting Information

S1 Table Statistical analysis of starvation survival from Fig 1A. The average half-life of biological replicates is reported along with the standard error of the mean (SEM) and the results of a t-test comparing each genotype to wild type or *daf-16* ^*null*^ without correction for multiple testing. p-values below 0.05 are in bold. At least three biological replicates were included for each genotype. The goodness of fit statistic R^2^ is reported for each genotype.
(PDF)

S2 Table t-test results for Fig 1B. The p-values from unpaired t-tests of the displayed pairwise comparisons are shown without adjustment for multiple testing. p-values less than 0.05 are in bold.
(PDF)

S3 Table Statistical analysis of starvation survival from Fig 5. The average half-life of biological replicates is reported along with the standard error of the mean (SEM) and the results of a t-test comparing each genotype to wild type or *daf-16* ^*null*^ without correction for multiple testing. p-values below 0.05 are in bold. At least three biological replicates were included for each genotype. The goodness of fit statistic R^2^ is reported for each genotype. The first two groups of strains (Fig 5A and 5B) included the P*hlh-8*::GFP reporter in the background. The *dbl-1*/TGF-β mutant displayed a nominally significant (p = 0.049) reduction of survival compared to wild type. However, the variation among *dbl-1* replicates is exceptionally small and seems to have led to a spurious p-value. The *sma-9*/co-SMAD mutant shows no effect (p = 0.72), supporting this conclusion.
(PDF)

S1 Fig Starvation survival with tissue-specific *daf-16*/FOXO rescue. The proportion of larvae surviving after seven days of starvation is plotted. Combined data from three independent extrachromosomal array lines and two biological replicates is plotted for each tissue-specific rescue. Significant differences among alleles for a given promoter or promoter pair were not observed. **p<0.01, *p<0.05; unpaired t-test against *daf-16* ^null^.
(TIF)

S2 Fig Allele-specific tissue-specific rescue of M cell division defect in *daf-16* ^*null*^. The proportion of larvae with at least one M lineage division after seven days of starvation is plotted along with the SEM. Each bar represents an independent transgenic allele and three to six biological replicates.
(TIF)

S3 Fig Visualization of L1 seam cell development using AJM-1::GFP. Fed wild-type L1 larvae expressing AJM-1::GFP are shown over developmental time focusing on a superficial focal plane to reveal seam cells (v1–6) on one side of the animal. (A) At the beginning of L1 development, v1–6 are visible in their undivided state with a distinct tapered shape. P cell membranes are also visible ventral to v1–6. (B) During mid-L1 development seam cells divide. Here three of them have recently divided (arrowheads), producing a transient total of nine seam cells. (C) After division, the anterior daughter of each seam cell fuses with the hypodermal syncytium, losing its membrane and AJM-1::GFP signal, and leaving the seam. Three fusing daughters are labeled with an asterisk. As a result, the remaining posterior daughters retain signal but are out of contact with one another. (D) The posterior daughters subsequently elongate and make contact with one another (arrows), reforming a seam and distinct appearance characterized by a rectangular shape and enlarged point of contact compared to the undivided seam cells in (A). Seam cells undergo an additional round of divisions near the L1 molt which is not shown here.
(TIF)

S4 Fig Insulin/IGF signaling is unlikely to mediate cell-nonautonomous effects of *daf-16*/FOXO on developmental arrest. The proportion of larvae with at least one M lineage division after seven days of starvation is plotted. Error bars reflect SEM of three-seven biological replicates. **p≤0.01; unpaired t-test against *daf-16*.
(TIF)

S5 Fig NanoString nCounter validation of candidate signaling genes identified with mRNA-seq. Transcript abundance during L1 arrest is plotted in arbitrary units for *daf-36*, *daf-12* and *dbl-1* for three temperatures and five genotypes. Counts for *daf-36* are generally below what is deemed reliable for this assay with the mass of RNA used for hybridization [55], though the effect of *daf-16* ^*null*^ is marginally significant. *daf-36* was also analyzed in independent samples by qRT-PCR and found to be significantly affected by *daf-16* ^*null*^ (see text). p-values for *daf-36*, *daf-12* and *dbl-1* (unpaired t-test pooling all replicates and temperatures) comparing wild type to *daf-16* were 0.04, 0.001 and 0.003, and comparing *daf-2(e1370)* to *daf-16; daf-2(e1370)* were 0.002, 0.001 and 0.006, respectively.
(TIF)

S1 Dataset mRNA-seq analysis of *daf-16*/FOXO during L1 arrest. Results of mRNA-seq analysis, including counts per gene, fold-changes, p-values adjusted for multiple testing (FDR), comparison with the meta-analysis of Tepper *et al* [9], and complete results of GO term enrichment analysis are included.
(XLSX)
