# Supplementary material for: dbl-1/TGF-β and daf-12/NHR Signaling Mediate Cell-Nonautonomous Effects of daf-16/FOXO on Starvation-Induced Developmental Arrest
Source: PLoS Genet. 2015 Dec 11;11(12):e1005731. doi: 10.1371/journal.pgen.1005731 (PMC4676721; doi:10.1371/journal.pgen.1005731)
Supplement: S1 Table — The average half-life of biological replicates is reported along with the standard error of the mean (SEM) and the results of a t-test comparing each genotype to wild type or daf-16 null without correction for multiple testing. p-values below 0.05 are in bold. At least three biological replicates were included for each genotype. The goodness of fit statistic R2 is reported for each genotype. (PDF) [file pgen.1005731.s001.pdf]

S1 Table. Starvation survival analysis of Figure 1A.

Genotype	Half-life	SEM	p-value (vs. wild type)	p-value (vs. *daf-16*)	R^2
wild type	10.5	0.58	N/A	**$3.8x10^{-6}$**	0.966
daf-16	4.7	1.01	**$3.8x10^{-6}$**	N/A	0.996
daf-16; P*daf-16*::DAF-16	10.8	1.15	0.681	**$6.7x10^{-5}$**	0.961
daf-16; P*ges-1*::DAF-16	9.1	0.76	**0.013**	**$5.0x10^{-5}$**	0.972
daf-16; P*unc-119*::DAF-16	7.2	1.34	**0.002**	**0.014**	0.986
daf-16; P*col-12*::DAF-16	8.6	1.26	**0.025**	**0.003**	0.987
