# Supplementary material for: dbl-1/TGF-β and daf-12/NHR Signaling Mediate Cell-Nonautonomous Effects of daf-16/FOXO on Starvation-Induced Developmental Arrest
Source: PLoS Genet. 2015 Dec 11;11(12):e1005731. doi: 10.1371/journal.pgen.1005731 (PMC4676721; doi:10.1371/journal.pgen.1005731)
Supplement: S2 Table — The p-values from unpaired t-tests of the displayed pairwise comparisons are shown without adjustment for multiple testing. p-values less than 0.05 are in bold. (PDF) [file pgen.1005731.s002.pdf]

S2 Table. t-test results matrix for Figure 1B.

		Rescue 2							
		P*unc-119* + P*col-12*	P*ges-1* + P*col-12*	P*unc-119* + P*ges-1*	P*myo-3*	P*col-12*	P*ges-1*	P*unc-119*	P*daf-16*
Rescue 1	no rescue	**0.001**	**0.013**	**0.001**	0.245	**0.003**	**0.010**	**0.001**	**$9.6x10^{-7}$**
	P*daf-16*	**0.003**	**0.008**	**0.005**	**$1.3x10^{-12}$**	**0.001**	**0.001**	**0.008**	N/A
	P*unc-119*	0.276	N/A	0.402	**$4.5x10^{-7}$**	0.521	0.302	N/A	
	P*ges-1*	N/A	0.596	0.090	**$2.5x10^{-5}$**	0.663	N/A		
	P*col-12*	0.088	0.845	N/A	**$2.7x10^{-6}$**	N/A			
	P*myo-3*	N/A	N/A	N/A	N/A				
	P*unc-119* + P*ges-1*	N/A	N/A	N/A					
	P*ges-1* + P*col-12*	N/A	N/A						
	P*unc-119* + P*col-12*	N/A							
