# Supplementary material for: dbl-1/TGF-β and daf-12/NHR Signaling Mediate Cell-Nonautonomous Effects of daf-16/FOXO on Starvation-Induced Developmental Arrest
Source: PLoS Genet. 2015 Dec 11;11(12):e1005731. doi: 10.1371/journal.pgen.1005731 (PMC4676721; doi:10.1371/journal.pgen.1005731)
Supplement: S3 Table — The average half-life of biological replicates is reported along with the standard error of the mean (SEM) and the results of a t-test comparing each genotype to wild type or daf-16 null without correction for multiple testing. p-values below 0.05 are in bold. At least three biological replicates were included for each genotype. The goodness of fit statistic R2 is reported for each genotype. The first two groups of strains (Fig 5A and 5B) included the Phlh-8::GFP reporter in the background. The dbl-1/TGF-β mutant displayed a nominally significant (p = 0.049) reduction of survival compared to wild type. However, the variation among dbl-1 replicates is exceptionally small and seems to have led to a spurious p-value. The sma-9/co-SMAD mutant shows no effect (p = 0.72), supporting this conclusion. (PDF) [file pgen.1005731.s003.pdf]

S3 Table. Starvation survival analysis of Figure 5.

Genotype	Half-life	SEM	p-value (vs. wild type)	p-value (vs. *daf-16*)	R^2
wild type	21.9	1.1	N/A	**0.002**	0.976
dbl-1	18.8	0.2	**0.049**	**0.002**	0.945
sma-9	22.3	0.7	0.720	**0.001**	0.970
daf-16	11.5	1.1	**0.002**	N/A	0.988
daf-16; dbl-1	9.7	1.3	**0.002**	0.355	0.976
daf-16; sma-9	9.9	1.1	**0.001**	0.371	0.951
wild type	21.2	0.8	N/A	**0.002**	0.980
daf-36	20.1	1.0	0.298	**0.004**	0.965
daf-9	24.9	0.5	0.060	**3.2×10^{-4}**	0.985
daf-12(rh273)	17.9	1.0	0.090	**0.002**	0.994
daf-16	11.6	0.6	**0.002**	N/A	0.986
daf-16; daf-36	13.2	0.9	**0.003**	0.263	0.959
daf-16; daf-9	11.9	1.5	**0.005**	0.805	0.981
daf-16; daf-12(rh273)	9.9	1.4	**0.001**	0.345	0.992
wild type	21.4	2.0	N/A	**0.011**	0.974
din-1	22.9	1.1	0.538	**0.002**	0.966
daf-12(m20)	21.4	2.0	0.987	**0.010**	0.977
daf-12(rh61rh411)	19.4	2.3	0.542	**0.032**	0.962
daf-16	11.2	1.1	**0.011**	N/A	0.977
daf-16; din-1	11.4	0.9	**0.010**	0.868	0.956
daf-16; daf-12(m20)	7.9	0.8	**0.003**	0.073	0.985
daf-16; daf-12(rh61rh411)	12.6	1.3	**0.020**	0.451	0.911
